# Notes on the Bacteriological Investigations of Wounds of the Soft Parts: Pratical Indications for Primary Suture

**Published:** 1918-03

**Authors:** L. Vaucher


### NOTES ON THE BACTERIOLOGICAL INVESTIGATIONS OF WOUNDS OF THE SOFT PARTS: PRATICAL INDICATIONS FOR PRIMARY SUTURE

L. VAUCHER

### The Development of Bactéria in the Wound.

If we study the development of bacteria in a wound, we are struck by the fact that the pullulation of organisms is not immediate. Between the moment of the contaminating injury and the beginning of the infection there is always a period the length of which depends on the depth and importance of the muscular injuries and on the amount of blood discharges into the contaminated wound.

The length of this period seldom exceeds 6 to 8 hours, but there are naturally great variations according to the type of wound.

The reality of such a period is proved:

1. By bacteriological investigation of the smears of the wound.
2. By histological investigation of the surrounding muscle.

*1. Smears of Fresh Wounds.* — The simple smear of a fresh wound in the very beginning shows pure blood, with some muscular tissue and very few or no organisms.

After six or eight hours the nature of the exudate changes. Instead of pure blood there is an important polymorphonuclear reaction. The organisms are numerous. Thick, long, grampositive bacilli, mostly without spores, are present and at the same time numerous cocci may develop but always less abundantly in the beginning than the bacilli.

*2. Histological Investigations.* — These investigations have shown that around the devitalized zone of muscle there is a more or less important zone infiltrated with blood. Only after six or eight hours do we notice a reaction of the tissue surrounding the wound; this reaction is characterized in the beginning by dilation of the vessels and by polymorphonuclear infiltration in the vessels and between the muscular fibers.

In short, bacteriology and histology give the same results. These two methods are equally clear in demonstrating that the surgical excision must be made soon, before the 6th or 8th hour, and that the later the operation the larger must be the excision with the knife.

Before the infection has spread it is possible to excise surgically not only the wound with its contents, — misssile, bits of clothing, clots etc. — but also the surrounding dead muscle which is an excellent culture medium for bacteria. If this excision is completely made, the logical conclusion is to proceed to the immediate suture of the wound.

### The Cultural Investigation.

The culture is the only bacteriological method which gives a precise answer revealing the nature of the aerobic and anaerobic bacilli contained in a wound.

If a bacteriologist is not especially familiar with the flora existing in war wounds it is necessary for him in the beginning to accomplish the complete bacteriological isolation of all aerobic as well as anaerobic microbes. This isolation is difficult and needs great care and much time. We have often been obliged to give the surgeon a quick answer; therefore we have used a simplified technique which, if not absolutely precise and scientific, gives satisfactory practical results in most cases. If, however, we want to know exactly how many different sorts of anaerobic bacilli exist in the wound, this simplified technique is unsatisfactory, but in most cases the answer which we are able to give to the surgeon is sufficiently precise and therefore we employ this method in times of great activity.

*Practical Method.* — 5 tubes are wanted for the investigation, 2 tubes of milk, 1 tube of broth, 2 tubes of slanted agar. This number can easily be brought down to two if the same wound must be often investigated, one tube of milk, and one of broth.

The specimens are collected in pipettes, the tubes of milk and broth are heavily inoculated; the tubes of slanted agar, on the contrary, are stretched lightly without loading the platinum loop again so as to obtain isolated colonies.

The milk must be heated for twenty minutes in the water bath so as to expel the air before inoculating the tube. It is possible to obtain the growth of anaerobic bacilli by adding to the milk a pinch of sulphide of calcium, but it is better to stretch the upper part of the tube and to seal it afterwards in the flame of a blowpipe.

I shall not speak of the cultural reactions and of the characteristics of the different types of organisms. I shall only insist upon the fact that milk is perhaps the best medium for anaerobic bacilli; it is surely the medium on which they present their most conspicuous characteristics.

Broth is a perfect medium for the streptococcus, it may develop in from 6 to 7 hours, before all of the other microbes of the wound. Slanted agar is the best medium for obtaining isolated colonies.

It is quite certain that by this practical method a number of the important anaerobic bacilli cannot be discovered. Those most commonly found are perfringens, sporogenes, and Henry's bacillus tertius (Von Hibler IX), Bacillus œdematiens, fallax, histolyticus, aerofetidus (Weinberg et Séguin). Vibrion septique and Bacillus tetani must be searched for by quite a different technique[1].

Nevertheless, as we are dealing with a great number of wounded, the method described above will be sufficient in nearly all cases, to indicate whether a wound may or may not be closed.

### The Bacteriological Investigation in the Primary Suture.

When the wound has been completely excised with the knife and hemostasis carefully performed, it is then sutured almost completely; there is left in the wound either a small drain or a bundle of horse-hair.

This technique has a double advantage :

1. The blood and serosity resulting from the muscular section do not remain in the wound.

2. The bacteriological investigations of the wound may be carried on.

In the beginning of our researches the specimens were taken from the wound itself with sterile instruments before the suture of the muscles. A certain number of small bits of muscles taken in different parts of the wound were placed directly in milk or in broth. The only advantage afforded by this method of procedure was the early answer (sometimes within 6 or 7 hours) which could be given in certain cases.

It is easier to study the serosity proceeding from the wound, aspired with a pipette from the depth of the wound after removing the drain or the bundle of horse hair. These may be continued in the wound until the bacteriological control is no longer necessary. The specimen must be obtained as soon as possible (within 6 to 10 hours after the suture). The liquid is examined on smears and cultivated as described above.

The smear often shows nothing but blood without polynuclear

1. The technique of isolation of the anaerobic bacilli will be described in detail in Weinberg and Seguin's book, *Gas Gangrene*, Paris, 1918.

cells or microbes. In other cases it shows numerous polynuclears or pus with gram-positive bacilli and cocci.

If numerous gram-positive bacilli are present, or chains of streptococci, the wound must be reopened at once[1].

The culture tubes will be examined at different stages, 6, 12, 24, 48 hours after the cultures have been taken.

We must know that often 6 hours after the culture, in cases where the wound is severely infected, it is possible, in broth, to discover chains of streptococci and, in milk, anaerobic bacilli may be found before the milk is clotted. Therefore one of the milk tubes may be opened at the 6th or 8th hour and the second left in the thermostat.

Certain results of the tests may be described as follows :

1. *When the cultures are completely sterile.* This fact may often be established 24 or 36 hours after the operation. All drainage either by tube or capillary must be immediately suppressed. It is possible to leave the wound shut with perfect security; if the wound is only three-quarters shut, which is sometimes a wise precaution, one or two supplementary sutures may be placed.

2. *When the culture shows the absence of anaerobes and of streptococcus.* Only some common aerobes — staphylococcus, colibacillus, Friedlander, or B. pyocyaneus are present. The complete shutting of the wound may be accomplished with perfect security.

3. *When the milk culture shows anaerobes* either pure or associated with aerobes, — staphylococcus, colibacillus, Friedlander, — but not with streptococcus. In this case the patient must be observed with the greatest care. The local state of the wound, and the general condition of the man are more important guides than the bacteriological examination.

Certain authors (Tissier for instance, whose contribution on the bacteriology of wounds is capital) state that really serious infectious accidents develop only when streptococcus is present, either alone or associated with anaerobes, and that all wounds which do not contain streptococcus can be shut with safety.

Our personal opinion differs on this point from Tissier's. In

1. The smear is a wholly unsatisfactory method. My personal opinion is that it is most dangerous to close a wound on the unconfirmed evidence of a smear. There is only one case in which the smear gives an really definite answer which indicates that the wound must be reopened immediately. That is when it shows numerous gram-positive bacilli, either alone or associated with chains of streptococci. This is a result which is frequently obtained and which necessitates the greatest precaution, because it is impossible from the smear to say which these bacilli are. For this information we must wait sometimes for 24 hours or more, for it can be obtained only by the culture. If such a wound is left closed a fatal gas gangrene may result.

three instances we have seen very serious accidents develop in patients whose wounds had been almost entirely sutured and contained only anaerobes associated with common microbes and no streptococcus. Moreover a great number of fatal cases of gas gangrene have been published in which the wound did not contain streptococcus. It is true that the association of streptococcus with anaerobes is a specially serious indication, but the absence of streptococcus does not surely indicate that a wound may be sutured primarily. The important publications of Weinberg and Séguin have demonstrated the high pathogenetic power of different anaerobes and specially the association of different sorts of anaerobes. This form of activity can be determined only by the clinical signs.

If the wound is in a good state, the skin loose without color, the limb neither edematous nor infiltrated by gas, and if the temperature is normal or moderate and the pulse not rapid, the drainage by tube or by bundle of horse hair must be left in the wound, but the wound must not be reopened.

If, on the contrary, the limb is edematous and contains gas, the skin red or bronzed, and the pulse rapid, even if the temperature is not very high, it is safer to reopen immediately and widely.

In a great number of cases the course to be followed is a question of clinical experience; it is not as bacteriologists but as clinicians that we must decide what to do.

4. There are cases in which the culture reveals streptococcus pure or associated with anaerobic bacilli. The wound must then be reopened widely.

*Is it possible by cultural reaction to distinguish among streptococci those that are really pathogenetic?*

The streptococcus often present in wounds is anaerobic as well as aerobic. It grows in broth without clouding the medium; it forms only tiny lumps; it clots the milk, does not redden the agar with litmus and lactose; and is strongly hemolytic. None of these characteristics is absolute. I think it dangerous to refuse to a gram-positive coccus the name of streptococcus because it does not present all of these characteristics. In the actual state of war surgery and war bacteriology it is safer to reopen a wound in which the culture reveals long chains of gram-positive cocci. Remember the fact that on some days the bacteriologist's work is enormous and that he cannot test all the characteristics of an organism. At times when the work is less intense, I strongly advise you to study a certain number of strains of streptococci isolated from wounds.

You will notice that among the characteristics two have really great value: the absence of disturbance in the broth, and the hemolytic power. The streptococci which are strongly hemolytic are habitually very pathogenetic, but the reverse is not always true.

These are the principal indications which may be given by the bacteriologist in the cases of primary suture. In short, there are wounds that must be sutured, these are the aseptic wounds and the wounds containing only aerobic microbes and no streptococcus. The wound containing streptococci must never be shut, and if shut. must immediately be reopened.

There are wounds which contain anaerobic bacilli and must be looked after very carefully. If the number of gram-positive bacilli is small the suture must not be completed : if it is great, it is wiser to reopen [1].

In fact these distinctions are often hypothetical; there are cases in which it is difficult for the bacteriologist to give an answer.

He must remember for instance, that among the wounds of the soft parts (those of the buttock, of the thigh — and especially the upper part of the thigh — and those of the calf of the leg) are particularly severe, because the muscular injuries are large as well as the blood effusion, and, if not properly treated, they give rise to gas gangrene. The bacteriologist must remember also that the injury of an important vessel makes the prognostic more serious. Furthermore, the examination of such a large wound must be carried out in a great number of places, and may give different results according to the place.

The investigation must therefore be repeated twice, even if the first results are negative, otherwise the surgeon may be led by the tests into a serious error. There is no danger to life in leaving a wound open 24 or 48 hours before suturing ; but a fatal gas gangrene in one shut too early is always to be feared.

### Bacteriological Examination of an Excised Wound when the Primary Suture is Postponed.

In times of great activity it is impossible to investigate bacteriologically the hundreds of wounds which pass through an ambulance. Moreover, the patients must be evacuated soon and cannot be kept under the prolonged care of the same surgeon. Therefore

1. Serious complications, particularly gas gangrene, are exceptional and are the result often of a bad surgical treatment (too late or incomplete). If gas gangrene occurs an immediate injection of a mixture of anti-perfringens, anti-vibrion-septique and anti-œdematiens sera must be made. It would be prudent in certain cases to inject preventively a mixture of the three sera. We adopted this method of procedure last summer in all wounds of the buttock, thigh, and calf of the leg, using the antitoxic sera prepared by M. Weinberg and Séguin. The results were excellent.

Pierre Duval has described what he calls the delayed primary suture. It is a wise way of proceeding because the accidents of primary suture are avoided. Besides, if the wound has been completely excised and only dressed with an aseptic dressing, it may remain practically sterile, and ready for suture during a certain number of days. We have been able to follow for 3 or 4 days wounds which have been carefully excised; the cultures showed that these wounds remained sterile under an aseptic dressing. The wounded, when properly excised, may be sent by train to a hospital situated far from the front and be sutured there as soon as possible.

The technique of the bacteriological test is the same as that described above. If the clinical aspect of the wound is perfect, one may, if necessary, take only a smear. But I do not advise this procedure because it gives a false security in certain cases and may lead to failure. The culture must be made in all the cases in which the clinical aspect is not perfect and the specimens should be taken from parts suspected of infection. It is sometimes wise to take the specimens from different parts of the same wound. It is also wise to wait 24 to 48 hours before giving the answer; the indications and contra-indications for suturing being the same as described above.

The great advantage of primary suture, either immediate or delayed, is that the scar will be supple and non-adherent because the suture has been performed on muscles and skin not infected and not granulating: the histological investigation of such scars shows very little or no fibrous tissue : on the contrary a wound which is cicatrized after long suppuration, either spontaneously or surgically, affords a rigid adherent scar entirely composed of fibrous tissue.

